# Preoperative humeral computed tomography may be a useful indicator of low bone mineral density in patients undergoing rotator cuff repair

**DOI:** 10.1016/j.xrrt.2025.100624

**Published:** 2025-12-01

**Authors:** Yuki Matsui, Yusuke Menjo, Yoshihiro Hojo, Daisuke Momma, Norimasa Iwasaki

**Affiliations:** a Department of Orthopaedic Surgery, Kushiro Rosai Hospital, Kushiro, Japan; b Department of Orthopaedic Surgery, Faculty of Medicine and Graduate School of Medicine, Hokkaido University, Sapporo, Japan; c Center for Sports Medicine, Hokkaido University Hospital, Sapporo, Japan

**Keywords:** Rotator cuff tear, Osteoporosis, Computed tomography, Dual energy X-Ray absorptiometry, Hounsfield unit, Proximal humerus, Bone mineral density, Multivariate linear regression analysis

## Abstract

**Background:**

Bone fragility can compromise rotator cuff repair, particularly in patients with osteoporosis. Although previous studies have demonstrated correlations between systemic bone mineral density (BMD) and bone quality, only a few have focused on the anchor-relevant regions in patients with rotator cuff tears. We hypothesized that preoperative computed tomography (CT)-based Hounsfield unit (HU) values at anchor-relevant areas in the proximal humerus could be used to estimate systemic BMD in these patients.

**Methods:**

This retrospective study included 58 patients (62 shoulders) who underwent arthroscopic rotator cuff repair and had both preoperative shoulder CT and dual energy X-ray absorptiometry scans. HU values were measured in 4 regions of interest within the proximal humerus: medial, central, lateral, and greater tuberosity (GT). Systemic BMD was assessed using femoral neck and lumbar spine T-scores and BMD values. Pearson correlation coefficients were used to evaluate the association between the HU values and systemic T-score/BMD. Multivariate linear regression analyses were performed using HU values at the GT after adjusting for age, sex, and rotator cuff tear size.

**Results:**

HU values were significantly lower in the lateral and GT regions than in the medial and central regions (*P* < .01). Among regions, the HU values at the GT showed the highest correlations with the systemic T-scores and BMD (all moderate): femoral neck T-score (r = 0.57), lumbar spine T-score (r = 0.55), femoral neck BMD (r = 0.60), and lumbar spine BMD (r = 0.56) (all *P* < .01). Multivariate regression confirmed that GT values were independently associated with both femoral and lumbar T-score/BMD (adjusted R^2^ = 0.37-0.45, *P* < .001), even after controlling age, sex, and tear size.

**Conclusion:**

Preoperative CT-derived HU values, particularly at the GT, have a moderate correlation with systemic T-scores and BMD in patients with rotator cuff tears. Given the lower HU values observed at the anchor insertion sites (GT and lateral regions), clinicians should consider the risk of bone fragility during surgical planning.

Rotator cuff tears are common among middle-aged and elderly individuals. Previous studies have reported a prevalence of 20.7%,^28^ increasing with age.^20^^,^^24^^,^^27^ Many patients in this age group also have coexisting osteoporosis.^10^^,^^11^^,^^26^

The shoulder is a non–weight-bearing joint, and a previous report demonstrated that bone mineral density (BMD) in the proximal humerus is significantly lower than that in weight-bearing joints such as the hip.^8^ Moreover, in patients with rotator cuff tears, stress transmission to the insertion site decreases, leading to reduced bone density, especially at the greater tuberosity (GT).^19^^,^^21^

Suture anchors are often used on the proximal humerus during rotator cuff repair. In osteoporotic bones, there is a risk of anchor pull-out or insufficient fixation during or after surgery. A significant positive correlation between the BMD and anchor pull-out strength has been reported.^16^^,^^25^ In addition, patients with osteopenia and osteoporosis have four- and seven-fold higher rates of postoperative retears, respectively.^2^

Therefore, preoperative evaluation of bone quality in the proximal humerus is clinically important, as it may influence surgical outcomes. However, Dual Energy X-ray absorptiometry (DEXA)-based BMD assessment is not routinely performed for all patients with rotator cuff tears, and no therapeutic interventions are currently provided.^5^^,^^11^ In contrast, depending on institutional practice and clinical indications, preoperative computed tomography (CT) is sometimes obtained for morphological assessment of the shoulder. Several studies have shown that Hounsfield unit (HU) values obtained from CT scans can be used to estimate BMD in various skeletal regions.^17^^,^^22^^,^^23^ Similar associations have been reported for the proximal humerus.^14^^,^^18^^,^^29^ Nevertheless, in patients with rotator cuff tears, the HU values of the cancellous bone in the proximal humerus may be reduced due to disuse and altered stress distribution,^3^ raising questions about whether HU values accurately reflect bone quality in this population. Furthermore, although anchor placement is critical during repair, few studies have evaluated HU values specifically at anchor insertion sites.

Therefore, it is important to evaluate whether preoperative HU values at the proximal humerus, specifically in regions corresponding to anchor placement, reflect systemic BMD in patients with rotator cuff tears. Although local bone density at anchor sites may be clinically relevant, the present study focused on systemic BMD due to feasibility constraints.

We hypothesized that preoperative proximal humeral HU values at anchor-relevant regions would correlate with systemic BMD as measured by DEXA. This study aimed to clarify the relationship between HU values in anchor-relevant areas of the proximal humerus and systemic BMD as measured by DEXA.

## Materials and methods

This retrospective study was approved by the institutional review board of our hospital. The requirement for informed consent was waived due to the retrospective nature of this study.

Patients who underwent surgical treatment for rotator cuff tears at our department between April 2023 and November 2024 were included. Those who underwent both preoperative shoulder CT and DEXA for BMD assessment were selected for analysis. The patients' average age, sex, and rotator cuff size were recorded, and tear size was classified according to previous reports.^4^^,^^7^

**Figure 1:**
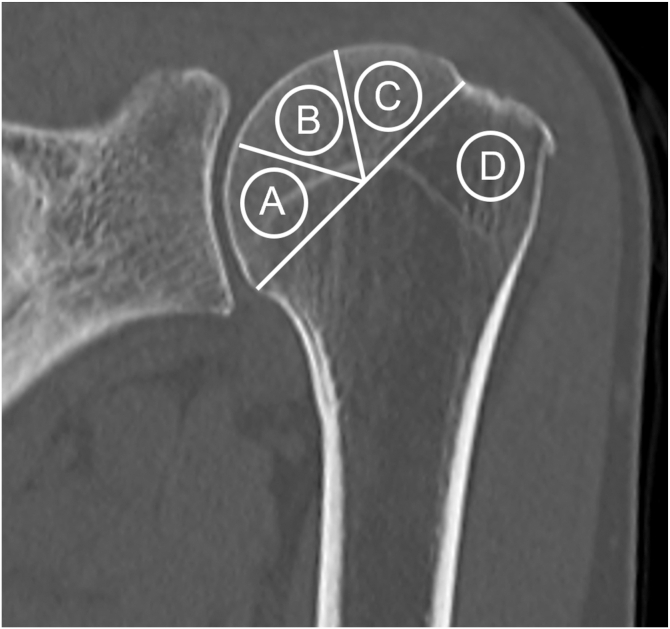
ROI in the proximal humerus. Four ROIs were defined on the coronal CT images: the medial, central, lateral, and greater tuberosity, which were measured within a standardized circular ROI (50 mm^2^) at each location. *ROIs*, regions of interest; *CT*, computed tomography.

The exclusion criteria were revision surgery, patients already undergoing treatment for osteoporosis, and those without preoperative CT or DEXA evaluations. In accordance with previous reports,^18^ regions of interest (ROIs) within the proximal humerus were defined at trabecular sites: medial, central, and lateral regions of the humeral head, and the GT, a typical lateral anchor insertion site during rotator cuff repair (Fig. 1). CT scans were then performed with patients in the supine position using a multidetector CT scanner (Aquilion Prime SP; Canon Medical Systems, Otawara, Japan) at 120 kVp with a slice thickness of 1.0 mm. All CT data were digitally archived and analyzed using a picture archiving and communication system and corresponding picture archiving and communication system software (XTREK View; J-MAC SYSTEM, INC, Sapporo, Japan). For each ROI, the area was set to 50 mm^2^, and the mean HU values were recorded.

BMD and T-scores at the femoral neck and lumbar spine were measured using DEXA (Horizon X; HOLOGIC Inc., Marlborough, MA, USA). The interval between shoulder CT and DEXA examinations was on average 21.2 ± 7.1 days, with a maximum of 30 days. These values were then used to evaluate their correlation with HU values from the four proximal humeral regions.

## Statistical analysis

Differences in CT attenuation values among the 4 ROIs (medial, central, lateral, and GT) were assessed using one-way analysis of variance. When significant differences were found, Tukey honest significant difference test was applied for post hoc pairwise comparisons. Univariate correlation analyses between HU values and BMD or T-scores were conducted using Pearson correlation coefficients.

Multivariate linear regression analyses were performed to investigate the association between the HU value at the GT and systemic BMD or T-scores of the lumbar spine and femoral neck. Age, sex, and rotator cuff tear size were included as covariates in regression models. Among the four CT measurements sites, the GT was selected for multivariate regression analysis based on its highest univariate correlations with BMD and T-scores.

Statistical significance was set at *P* < .05 all tests. Statistical analyses were performed using EZR (ver. 1.68; Saitama Medical Center, Jichi Medical University, Saitama, Japan), a graphical user interface for R (The R Foundation for Statistical Computing, Vienna, Austria). Post hoc power analyses were conducted for each regression model using G∗ Power (version 3.1.9.7; Heinrich Heine University Düsseldorf, Düsseldorf, Germany), with effect sizes calculated based on adjusted R^2^ values.

## Results

Among the 69 patients (71 shoulders) who underwent surgical repair of rotator cuff tears, 58 (62 shoulders) who underwent both preoperative shoulder CT and DEXA measurements were included in the analysis. The mean age was 68.2 ± 8.0 years (range, 49-82 years), and the cohort consisted of 37 males and 23 females. Rotator cuff tear size was classified as partial-thickness tears in 9 shoulders, small tears in 3, medium tears in 21 shoulders, large in 6, and massive tears in 20 shoulders, according to the established criteria.^4^^,^^7^

**Figure 2:**
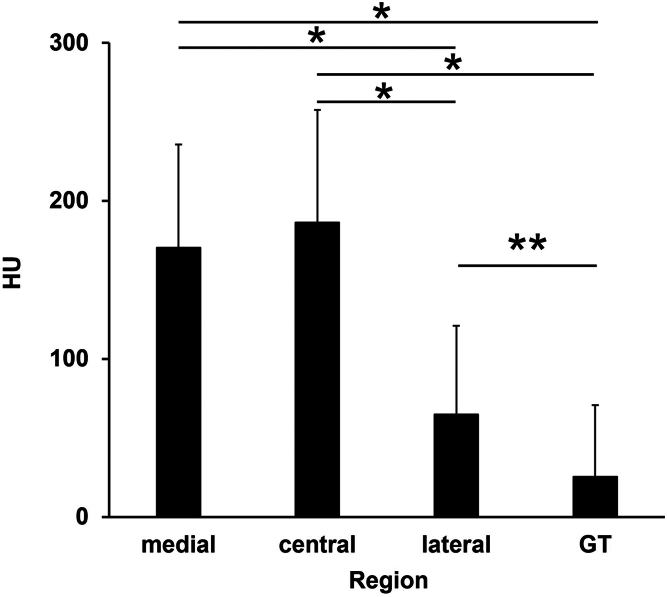
Comparison of HU values among the 4 regions of interest in the proximal humerus. The HU values in the lateral and GT regions were significantly lower than those in the medial and central regions. The GT region was also significantly lower than the lateral region. Error bars represent the standard deviation. Statistical significance was determined using one-way ANOVA followed by Tukey post hoc test; ∗*P* < .01. ∗∗*P* = .02. *GT*, greater tuberosity; *HU*, Hounsfield unit; *ANOVA*, analysis of variance.

The mean HU values at the proximal humerus were as follows: medial, 170.5 ± 65.3; central, 186.4 ± 71.2; lateral, 64.9 ± 56.1, GT, 25.4 ± 45.4. HU values were significantly lower in the lateral and GT regions than in the medial and central regions (Fig. 2).

The mean T-scores were −0.4 ± 2.0 at the lumbar spine and −1.0 ± 1.0 at the femoral neck. The corresponding BMD values were 1.0 ± 0.2 g/cm^3^ at the lumbar spine and 0.7 ± 0.1 g/cm^3^ at the femoral neck (Table I)

**Table I:** Patient demographics, mean CT attenuation, and DEXA measurements.

Variable	Mean ± SD or Number
Age (yr)	68.2 ± 8.0
Sex	
Male	37
Female	23
Rotator cuff tear size	
Partial	9
Small	3
Medium	21
Large	6
Massive	20
HU values	
Head-medial	170.5 ± 65.3
Head-central	186.4 ± 71.2
Head-lateral	64.9 ± 56.1
GT	25.4 ± 45.4
T-score	
Lumbar spine	−0.4 ± 2.0
Femoral neck	−1.0 ± 1.0
BMD	
Lumbar spine	1.0 ± 0.2
Femoral neck	0.7 ± 0.1

*HU*, Hounsfield unit; *BMD*, bone mineral density; *CT*, computed tomography; *DEXA*, Dual Energy X-ray absorptiometry; *GT*, greater tuberosity.

Pearson correlation analysis showed that lumbar spine T-score had its highest regional correlation with the HU value of the GT (moderate; r = 0.55, *P* < .001), with weaker positive correlations in the medial (r = 0.32, *P* = .01), central (r = 0.43, *P* < .001), and lateral (r = 0.37, *P* = .003) regions. The femoral neck T-score similarly correlated most with the GT HU value (moderate; r = 0.57, *P* < .001) and weaker correlations with the central (r = 0.32, *P* = .01) and lateral (r = 0.46, *P* < .001) regions.

**Figure 3:**
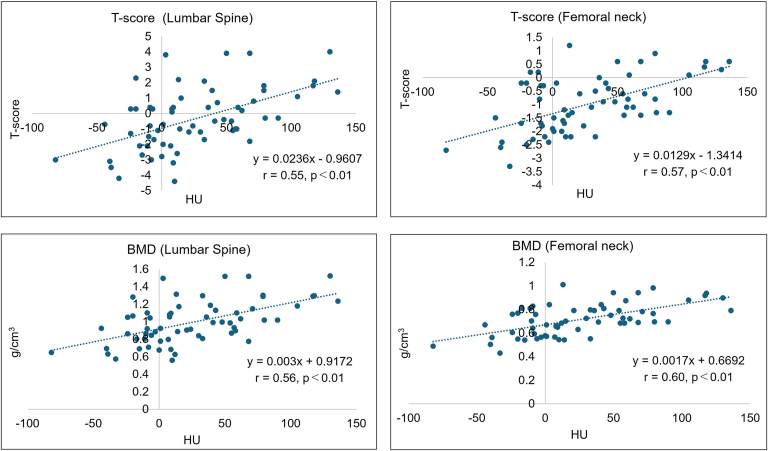
Scatter plots showing the correlations between HU values at the GT and systemic bone mineral status. HU values in the GT were positively correlated with T-scores and BMD at both the femoral neck and lumbar spine (Pearson correlation, *P* < .01 for all comparisons). *GT*, greater tuberosity; *HU*, Hounsfield unit; *BMD*, bone mineral density.

Similarly, lumbar spine BMD was positively correlated with HU values at the GT (moderate; r = 0.56, *P* < .001) and showed weaker positive correlations with the medial (r = 0.31, *P* = .013), central (r = 0.43, *P* < .001), and lateral (r = 0.37, *P* = .003) regions. The femoral neck BMD was significantly correlated with the HU value of the GT (moderate; r = 0.60, *P* < .001) and weakly correlated with the medial (r = 0.31, *P* = .01), central (r = 0.39, *P* = .002), and lateral regions (r = 0.49, *P* < .001) (Table II, Fig. 3).

**Table II:** Correlations between HU values and DEXA measurements (Pearson r).

HU values	BMD				T-score			
	Lumbar spine		Femoral neck		Lumbar spine		Femoral neck	
	r	*P* value	r	*P* value	r	*P* value	r	*P* value
Head-medial	0.31	.013	0.31	.01	0.32	.01	0.23	.07
Head-central	0.43	<.001	0.39	.002	0.43	<.001	0.32	.01
Head-lateral	0.37	.003	0.49	<.001	0.37	.003	0.46	<.001
GT	0.56	<.001	0.60	<.001	0.55	<.001	0.57	<.001

*HU*, Hounsfield unit; *BMD*, bone mineral density; *GT*, greater tuberosity; *DEXA*, Dual Energy X-ray absorptiometry.

Multivariate linear regression analyses revealed that the HU value of the GT was independently associated with both T-scores and BMD of the lumbar spine and femoral neck after adjusting for age, sex, and rotator cuff tear size. The adjusted R^2^ values were 0.37 (lumbar spine T-score), 0.40 (femoral neck T-score), 0.38(lumbar spine BMD), and 0.45 (femoral neck BMD), indicating moderate explanatory power. All models demonstrated statistically significant overall fit (F = 9.77-13.55, *P* < .001). No evidence of multicollinearity was observed (all variance inflation factors < 2). Detailed regression results, including β coefficients, 95% confidence intervals, and *P* values, are presented in Table III.

**Table III:** Multivariate linear regression analyses of the associations between CT values of the greater tuberosity and DEXA outcomes.

Variable	T-score lumbar spine β (95% CI)	*P* value	T-score femoral neck β (95% CI)	*P* value	BMD lumbar spine β (95% CI)	*P* value	BMD femoral neck β (95% CI)	*P* value
HU value (GT)	0.018 (0.007-0.028)	.001	0.013 (0.008- 0.018)	<.001	0.002 (0.001- 0.004)	<.001	0.001 (0.0008-0.002)	<.001
Age	0.027 (−0.025 to 0.080)	.303	0.006 (−0.021 to 0.033)	.660	0.003 (−0.003 to 0.010)	.299	0.001 (−0.002 to 0.004)	.543
Sex	−1.43 (−2.40 to −0.47)	.004	−0.268 (−0.760 to 0.223)	.279	−0.172 (−0.289 to −0.055)	.005	−0.085 (−0.145 to 0.025)	.006
Rotator cuff tear size	0.34 (−0.25 to 0.93)	.254	0.456 (0.155- 0.756)	.004	0.036 (−0.035 to 0.108)	.313	0.045 (0.009- 0.082)	.016

*CI*, confidence interval; *HU*, Hounsfield unit; *GT*, greater tuberosity; *DEXA*, Dual Energy X-ray absorptiometry; *BMD*, bone mineral density; *CT*, computed tomography.

## Discussion

In this study, we investigated the correlation between proximal humeral HU values and systemic BMD in patients with rotator cuff tears. T-scores and BMD values of the lumbar spine and femoral neck showed significant positive correlations with HU values in multiple regions of the proximal humerus, with the highest association observed at the GT. Based on these findings, we conducted multivariate linear regression analyses using HU values at the GT, adjusting for age, sex, and rotator cuff tear size as potential confounders. Even after the adjustment, the association remained statistically significant. In addition, the HU values were significantly lower in the lateral and GT regions than in the medial and central regions of the humeral head.

Although previous studies have demonstrated correlations between femoral or spinal BMD and local CT-derived HU values,^17^^,^^22^^,^^23^ only a few have specifically examined the proximal humerus in patients with rotator cuff tears. Lee et al^18^ reported a correlation between the central region of the humeral head HU values and BMD. Similarly, Kirchhoff et al^13^ used high-resolution quantitative CT of cadaveric shoulders and identified the posteromedial aspect as having the highest bone quality. This region corresponds anatomically to the central region analyzed in our study, supporting the consistency of our findings.

Suture bridge techniques have become increasingly popular for rotator cuff repair.^6^^,^^9^ In these procedures, the lateral portion of the humeral head is typically used for medial anchor placement, whereas the GT is used for lateral anchors. Preoperative CT imaging may not only aid in morphological assessment but also allow the estimation of bone quality by measuring HU values at the GT.

Several studies have suggested an association between osteoporosis treatment and improved outcomes after rotator cuff repair. Cancienne et al^1^ reviewed 2,706 patients undergoing arthroscopic rotator cuff repair and found that those with osteoporosis had a significantly higher reoperation rate than those without osteoporosis (6.58% vs. 4.51%). Lee et al^15^ reported that intravenous zoledronic acid reduced the retear rate (13.3% vs. 25%). Similarly, Zhao et al^30^ demonstrated that zoledronic acid significantly decreased retear rates in patients with osteoporosis (30.3% vs. 13.3%). Kim et al^12^ found that treatment with denosumab reduced the retear rate to a level comparable to that in nonosteoporotic patients (16.7% vs. 11.7%).

When low BMD is suspected based on preoperative CT findings, further evaluation using DEXA, serum bone metabolism markers, and thoracolumbar spine radiographs may be beneficial for diagnosing osteoporosis. If osteoporosis is diagnosed, initiating appropriate treatment before surgery may help reduce the risk of rotator cuff retears.

This study had several limitations. First, we did not directly measure proximal humeral site-specific BMD. HU values were obtained from noncalibrated clinical CT without phantom-based calibration or conversion to BMD, and we did not use other local BMD modalities such as peripheral quantitative computed tomography or humeral DEXA. Nevertheless, the observed moderate correlations between proximal humeral HU and systemic BMD suggest potential opportunistic utility. Future studies comparing local and systemic bone measures and linking them with surgical outcomes will be essential to clarify the clinical relevance of these findings. Second, we did not analyze surgical outcomes; therefore, any clinical implications should be viewed as hypothesis-generating. Third, the study was conducted at a single center with a relatively small sample size. Further multicenter studies with larger populations are required to validate our findings. Fourth, only the central region of the humeral head was evaluated on the sagittal plane. Nevertheless, significant correlations between the HU values at this location and systemic BMD were observed, supporting the adequacy of this single-plane measurement. Finally, CT and DEXA were performed within 30 days for all included shoulders, but we did not compute summary interval statistics, which should be addressed in future work.

This study's strengths include the systematic assessment of 4 anatomically relevant regions in the proximal humerus, selected with anchor placement during arthroscopic rotator cuff repair in mind. In addition, multivariate analyses were performed with adjustment for potential confounding factors to improve the reliability of the observed associations between HU values, BMD, and T-scores. These findings provide a methodological basis for future investigations exploring the potential clinical implications of proximal humeral bone quality.

## Conclusion

In this study, we investigated the relationship between HU values at various regions in the proximal humerus and systemic BMD in patients with rotator cuff tears. T-scores and BMD values of the lumbar spine and femoral neck showed positive, moderate correlations with HU values at multiple regions of the proximal humerus, with the highest association found at the GT. Multivariate regression analysis confirmed that the HU value of the GT was independently associated with the systemic T-score and BMD. Moreover, the HU values in the lateral and GT regions were significantly lower than those in the medial and central regions. These findings suggest that lower HU values at anchor-relevant regions of the proximal humerus, particularly the GT, may indicate reduced systemic bone density, supporting their potential utility as opportunistic indicators of bone quality on preoperative shoulder CT.

## Acknowledgment

The authors would like to thank Editage (www.editage.com for English language editing.

## Disclaimers:

Funding: No funding was disclosed by the authors.

Conflicts of interest: The authors, their immediate families, and any research foundations to which they are affiliated have not received any financial payments or other benefits from any commercial entity related to the subject of this article.
